# “Kicking and Screaming” or “Gracefully Conceding”: Creative Nonfiction Stories of Aging With Multiple Sclerosis

**DOI:** 10.1177/10497323211009864

**Published:** 2021-04-30

**Authors:** Emma V. Richardson, Robert W. Motl

**Affiliations:** 1 University of Worcester, Worcester, United Kingdom; 2 University of Alabama at Birmingham, Birmingham, Alabama, USA

**Keywords:** aging, multiple sclerosis, experiences, qualitative, chronic illness, pluralistic analysis, creative nonfiction, United States

## Abstract

Aging with multiple sclerosis (MS) is a complex phenomenon. Some individuals report physical and cognitive dysfunctions regarding these combined experiences, whereas others report perceived improvements in quality of life. Beyond this, little is known regarding how people make sense of, and come to embody, negative or positive experiences of MS. Thus, our objectives were to (a) explore how people made sense of aging with MS and (b) present this in an artful, engaging, transformative way. To achieve this, we conducted 40 semi-structured interviews with older adults who had MS, analyzed data using pluralistic narrative analyses, and presented results through two creative nonfictions. We detail our process of creating the nonfictions before presenting the different stories of aging with MS, namely “Kicking and Screaming” and “Gracefully Conceding.” We then offer recommendations and implications for using these stories as knowledge translation devices, and further critique the limitations of these stories in practice.

## Introduction

Historically, the dominant age range of persons with multiple sclerosis (MS) has been between 45 and 55 years of age; however, the most recent MS epidemiological study indicated that the most populous age range is now between 55 and 65 years of age—a 10-year difference (Wallin et al., 2019). We are therefore seeing a demographic shift of an aging MS population. This “graying” phenomenon is a welcome advancement, and a testament to the increasing number of pharmacological (e.g., increases in the number of disease-modifying therapies) and rehabilitation milestones (e.g., clinically established exercise guidelines; Latimer-Cheung et al., 2013) that have been achieved over the past 30 years. For example, there were seven disease-modifying therapies primarily for relapsing–remitting MS in 2010 (Binks & Dobson, 2015) compared with 20 disease-modifying therapies for multiple courses of MS in 2020 (National Multiple Sclerosis Society [NMSS], 2020). This advancement, however, brings into question the combined effects of aging with MS. This can be partitioned into questions of, “what do persons with MS experience with age?” and “how do persons with MS make sense of these experiences?”

MS is caused by an inflammatory immune-mediated response in genetically susceptible people that causes the body’s immune system to attack myelin surrounding nerves in the central nervous system (i.e., brain, brain stem, spine, and optic nerves). The resulting damage causes lesions or scars that, depending on severity and location, cause numerous physical and psychological symptoms (Milo & Miller, 2014). These include, but are not limited to, cognitive, walking, sexual, visual, and bowel and bladder dysfunction, pain, spasticity, fatigue, anxiety, and depression (Motl, 2010; Motl et al., 2013; Sandroff et al., 2015). MS further manifests in different ways, either relapsing–remitting MS or progressive MS. Relapsing–remitting MS is the most common phenotype and is characterized by clearly defined periods of worsening symptoms followed by prolonged periods of cessation. Progressive MS is less common and characterized by gradual and continual worsening symptoms. Importantly, relapsing–remitting MS may transition into a progressive form (secondary progressive MS) over time with 50% of persons with relapsing–remitting MS transitioning to a progressive form within 10 years of diagnosis, and 90% transitioning to a progressive form within 25 years (NMSS, 2020). Aging, therefore, plays a pivotal role in the embodied experience of MS.

To date, research that has explored aging in MS has highlighted the complexity and contradictions of this experience. For example, some research described a “double impairment effect” in that the combined pathologies of aging and MS worsened physical function, cognitive function, dependence, mental health, emotional health, bowel and bladder function, comorbid conditions, and quality of life (E. V. Richardson & Motl, 2020; Sanai et al., 2016; Solaro et al., 2015). Other research proposed that, with age, the inflammatory phase of MS may “burn out” such that symptoms stabilize in later life (Hua et al., 2019). Alternatively, though experiencing physical and cognitive dysfunction, persons with a longer disease duration (e.g., more than 20 years) may have increased resilience and preparedness for aging with MS, as MS symptoms can mirror the aging process (Dilorenzo et al., 2008; Finlayson et al., 2005). Persons with MS may further experience a sense of normalcy and connection to aging peers as they share experiences of similar cognitive and physical dysfunctions (E. V. Richardson & Motl, 2020). The aforementioned works provide a grounding for the different ways aging with MS may be interpreted and experienced, but there is still a gap regarding *how* persons with MS make meaning and sense of aging with a chronic illness, and how they come to embody positive or negative experiences. Such exploration is essential due to the unpredictable disruption MS can play in a person’s life; there is a constant engagement in making sense of one’s embodied experiences and maintaining a sense of self (Taylor, 1983). Indeed, research has highlighted how people with MS make sense and meaning of an MS diagnosis (Isaksson & Ahlström, 2006), how to live with MS (Pakenham, 2007), and how to manage life with progressive MS (J. Frost et al., 2017). These works show invaluable insight regarding how persons with MS make sense of living with this condition. However, an exploration of how people with MS make sense and meaning of aging is still wanting. Our premise is that developing an appreciation of this experience may be of use to (a) health care providers who treat an ever-aging and populous MS demographic and (b) persons aging with MS as this information can translate knowledge of aging with MS and encourage connectedness, and validation of this lived experience. To do so, we utilized the qualitative tradition of narrative.

Human beings are story-telling creatures that make sense and meaning of their lives through narrative (Crossley, 2000). Narrative sensemaking “helps individuals organize lived events—many of which are messy, multivocal, complicated or confusing—into more manageable pathways that make sense of the context of their lives and relationships” (Koenig Kellas, 2015, p. 254). As aforementioned, engaging in sensemaking is a pivotal part of living with MS, as living with periods of relapses or continued deterioration requires continued engagement in narrative to maintain a sense of self. Indeed, narrative is a particularly effective method in the context of chronic illness as during times that our sense of self is endangered (e.g., during a relapse), our tendency is to turn to narrative to rework and reexamine our life story and maintain a sense of identity (Bury, 1982). The temporality of narratives is also important to consider regarding aging with MS as “the self” continually evolves throughout our lives as embodied experiences change through time (Charmaz, 1991). This is not only because we begin to tell stories about ill bodies, but aging bodies (Phoenix et al., 2010) as well as the cultural, social resources we use to interpret these experiences change. That is, we do not create stories out of thin air, but draw upon a cultural menu that shapes our personal experience (Phoenix & Sparkes, 2006). For example, the addition of a Western narrative of aging dominantly telling a story of physical and cognitive decline (Gullette, 2004) can change a person with MS perception of their own body from “dysfunction” to “normal” as they now align to cultural expectations of that stage of life (E. V. Richardson & Motl, 2020). Narrative also lends itself to aging with MS as this tradition can translate knowledge about complex phenomena by using common language and emotive techniques that may be more understandable and meaningful (Skains, 2018). For example, a narrative lens has illuminated persons with MS perceptions of exercise (E. V. Richardson et al., 2018) and masculinities (Riessman, 2003). Those works, however, were presented as realist tales and may have missed the emotive, multitextured, moving essences that stories can provide. Those works therefore missed an opportunity to get under one’s skin (Frank, 2010), giving a sense of belonging, validation, and empowerment as one sees oneself represented in a story. Therefore, in this work, we aim to “get under one’s skin” by *showing* what aging with MS can be like through the creative analytic practice (CAP) of creative nonfiction.

## Method

### Design and Philosophical Assumptions

CAP is an overarching term that represents different kinds of alternative, arts-based, and performative research practices as opposed to traditional scientific representations of research (L. Richardson, 2000). There are numerous types of research that are considered to be CAP, such as autoethnography, poetry, and ethnodrama, which are used to ensure the complexity of lived experience is captured (McMahon, 2016). For this particular study, we engaged in a creative nonfiction design. Creative nonfictions tell stories that are grounded in research data but (re)presented in a storied fashion:

Creative nonfiction tells a story using facts, but uses many of the techniques of fiction for its compelling qualities and emotional vibrancy. Creative nonfiction doesn’t just report facts, it delivers facts in ways that move the reader toward a deeper understanding of a topic. (Cheney, 2001, p. 1).

Past topics that have (re)presented knowledge this way include rehabilitation experiences of a spinal cord injury (Smith, 2013), the impact of social relationships for engaging in physical activity with a physical impairment (Javorina et al., 2020), active aging (Wright, 2019), and difficulties managing MS within a workplace (Vickers, 2014), but aging with MS has yet to be explored.

The purposes and effectiveness of using creative nonfiction for sharing knowledge of aging with MS are fourfold. (a) Creative nonfiction can capture the contrasting experiences of chronic illness, and evocatively and meaningfully show the interweaving and complex whats, whys, and hows of lived experience (e.g., see Nowakowski, 2016; Nowakowski & Sumerau, 2019): What did people experience aging with MS? How did people experience aging with MS? Why were different stories of aging told? (b) Creative nonfiction can ensure human conduct and lived experience is portrayed in a way that respects agency and structure (Gerard, 2017). (c) Our purpose as researchers is to serve the MS community and share our research with those that make our research possible (Vickers, 2014). (d) Creative nonfiction is accessible to a wider audience than academics, and is a highly effective knowledge translation technique (Griffin & Phoenix, 2014). This is evident in work, as one example, showing different barriers, facilitators, fears, emotions, and trajectories of spinal cord injury rehabilitation (Smith et al., 2013). There have been increasing calls within health disciplines to utilize methods to show the complexity of experiences, and to ground knowledge claims with practical application by using techniques that represent participants and can show the complexity of an experience to different audiences in an understandable way (Papoutsi et al., 2021). Thus, a creative nonfiction design provides a novel, meaningful, and accessible way to (re)present the lived experiences of aging with MS, and share this with multiple audiences.

To do a creative nonfiction, we utilized the “tips” proposed by Smith et al. (2015) to assist in our thinking regarding how to transform the data into a story, and the detailed, contextual guides of Orr et al. (2020) and Hall (2020). Smith et al. (2015) proposed the necessity to (a) have a purpose (which we presented through the introduction), (b) use analysis and theory to interpret the story, (c) show findings rather than tell findings, (d) ensure verisimilitude (truthfulness to the experience), (e) select and develop characters to tell the story, (f) use dialogue to bring findings to life, (g) show bodies being expressive and acting, (h) write evocatively, (i) develop a plot, (j) set the scene, (k) select which parts of the story need to be told, (l) think about the story with your body, (m) edit, and (n) be epistemologically and ontologically aware. These different requirements are apparent throughout our “Process of Constructing Nonfictions” section.

As a first port of call, we present out epistemological and ontological assumptions. The paradigmatic assumptions of this study were shaped by narrative constructionism. Narrative constructionism understands narratives as a vehicle by which worlds, lives, and selves can be better appreciated, articulated, experienced, and understood through one’s relationship with the self, others, and social worlds (Gergen, 1999). Narratives are stressed as sociocultural, relational phenomena (Smith & Sparkes, 2008a); that is, while people may have an embodied intuition of personal experiences, this intuition is constantly being reshaped when stories are shared among others, and circulated in culture and society. Accordingly, narrative constructionism is underpinned by ontological assumptions that human life is storied and a condition of social life (Burke, 2016), and epistemological assumptions that narrative is both a way of telling about our lives and a method of knowing (L. Richardson, 2000). This understanding of narrative facilitated the creation of two creative nonfictions reflecting the embodied, lived experiences of persons aging with MS.

### Sampling and Participants

The authors received ethical approval from the University of Alabama at Birmingham before the study began. We engaged in purposive sampling through electronic recruitment via the NMSS by advertising a qualitative study exploring experiences of aging and wellness in MS. More than 300 persons contacted the lab expressing interest. To manage this number of persons, and ensure rigor and quality data collection, we utilized criterion-based inclusion, and maximum variation techniques (Palinkas et al., 2015). The inclusion criteria were (a) age of 60 years or older, (b) confirmed diagnosis of MS, (c) fluent in English, and (d) willingness to take part in a recorded interview lasting between 1 and 2 hours. This approach allowed us to select persons that could speak to the research questions and provide in-depth experiential data regarding aging with MS. To gain a cultural cross-section of experiences, we sought equal representation of the North, South, East, and West of the United States. Thus, we selected 10 persons from each of these four areas (*n* = 40) and deliberately selected participants of different ages, disease durations, type of MS, and genders to gain an appreciation of a range of aging with MS experiences (Smith & Sparkes, 2016).

Overall, we selected 40 participants. Each participant was contacted and completed a telephone-based screen for inclusion criteria. If participants met the criteria and consented to participate, a day and time for the interview was arranged. Of the 40, 29 participants were female, and 11 were male. Age ranged between 60 and 85 years with a mean age of 67.5 years. Disease duration of MS ranged between 3 and 55 years with a mean disease duration of 25 years. Nineteen participants had relapsing–remitting MS, and 21 had progressive MS. Eighteen were ambulatory, 17 used a walking aid, and five used a wheelchair or powerchair. Thirty-three were retired, five were employed on a part-time basis, and two on a full-time basis.

### Data Collection

A semi-structured design was used to focus on areas of interest such as the experience of aging, messages of aging, and expectations of aging, but further provided some freedom and space to discuss other areas of interest that were relevant and important to each participant (Smith, 2016). Moreover, informed by our narrative constructionist paradigm, questions were crafted in such a way to encourage storied data and provide a space for sharing stories such as “Tell me a story about . . .” “Please tell me about a time when this happened” (the full interview guide is attached in Appendix 1). In this way, the researcher and participant could co-construct meaningful narratives of aging with MS. The interview guide was crafted through engagement with previous literature regarding aging with MS (e.g., Dilorenzo et al., 2008; Finlayson et al., 2005), a focus group at the opening of the Healthy Aging through LifesTyle in MS center, and methodology regarding how to craft a semi-structured interview guide (Sparkes & Smith, 2013). The guide was then further refined and used as a tool to guide the interview and ensure questions regarding aging with MS were asked. Notes were made throughout the interviews and assessments discussed between authors regarding whether the guide needed amending (e.g., a question added, changed, or removed). Notes of interview content were transferred to a word document when the interview was complete in-line with good interview practice.

We used video conferencing software to conduct the semi-structured interviews. This is the preferred method within MS qualitative research as it allows for managing fatigue, and reducing stress associated with travel; this can result in richer, more in-depth data and a more enjoyable experience for the participant (Synnot et al., 2014). We conducted all interviews, which began after participants gave verbal consent that recording could begin. Interviews ranged between 58 and 118 minutes with a mean interview length of 78 minutes. There was a total of 3,116 minutes (54.5 hours) of raw interview data. Participants received a US$50 visa gift card for participating. Raw audio data were sent to an external transcription company immediately after the interview. Upon receipt of the transcript, we removed names and identifying information, and checked for accuracy against the original recording.

### Process of Constructing Creative Nonfictions

We utilized Hall’s (2020) methodological process as an exemplar for *showing* how we constructed two creative nonfictions of aging with MS. We further drew upon Orr et al.’s (2020) reflexive piece “Café Talk” as an insightful resource for how to develop different aspects of the stories.

**Figure 1.**
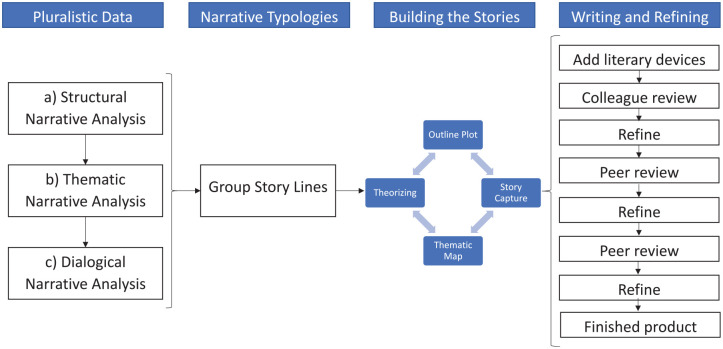
Creation nonfiction construction process.

Figure 1 represents our process for creating the two creative nonfictions, namely “Kicking and Screaming” and “Gracefully Conceding.” The following section describes and shows the step-by-step analytical and creative process we undertook to create these stories. In so doing, we show how we (a) analyzed the data, (b) applied theory and conceptual interpretation to the data, and (c) wrote the stories in such a way that they could stand alone as theory by themselves (Smith, 2013). To accomplish this, we transitioned between the lenses of story analysts and storytellers. Story analysts focus on the context of the stories (Gubrium & Holstein, 2009) and step outside of the story to apply analytical procedures to scrutinize, explain, and think about different features such as plot, themes, and effects (Smith & Sparkes, 2008b). Storytellers *show* stories through producing a tale, rather than discussing through a disembodied voice (Ellis & Bochner, 2006), that is artful, evocative, empathetic, multivoiced, and an engaging “way of knowing” about an experience (L. Richardson, 2000).

#### Pluralistic data analysis

From a story analyst lens, we first analyzed the interview scripts through analytical pluralism. Pluralistic data analyses involve the application of at least two different analytical techniques used on qualitative data to represent multiple aspects of a phenomenon (e.g., aging with MS; Clarke et al., 2015). Clarke et al. continued stating that through a pluralistic analytical method, more nuanced, rich, dynamic, and complex understandings can be crafted as different analytical lenses are applied. Furthermore, this approach can effectively represent the multidimensional, and potentially contrasting, aspects and interpretations of a phenomenon. In so doing, findings may be relevant and empowering to different audiences, for example, validating a person’s lived experience that may not be the dominant norm (N. Frost & Nolas, 2013). By presenting contrasting versions of the same phenomenon, different experiences can be given equal significance. This is particularly relevant for aging with MS as previous literature has highlighted alternate and contradicting embodied experiences such as aging making MS better and aging making MS worse (E. V. Richardson & Motl, 2020). By given equal significance to these contrasting experiences, rather than considered a “right” or “wrong” way, pluralism invites consideration that each story can be useful and purposeful (Honan et al., 2000). Generating these new ways of understanding can potentially empower readers through validating lived experience, and transform experiential knowledge into practical knowledge (Clarke et al., 2015) as stakeholders (such as persons with MS, family members, health care providers) learn more about different, valid interpretations of lived experiences. To do a pluralistic data analysis, we concurrently applied (a) structural narrative analysis, (b) thematic narrative analysis, and (c) dialogical narrative analysis (DNA), which were underpinned by our narrative constructionist paradigm.

##### Structural narrative analysis

**Figure 2.**
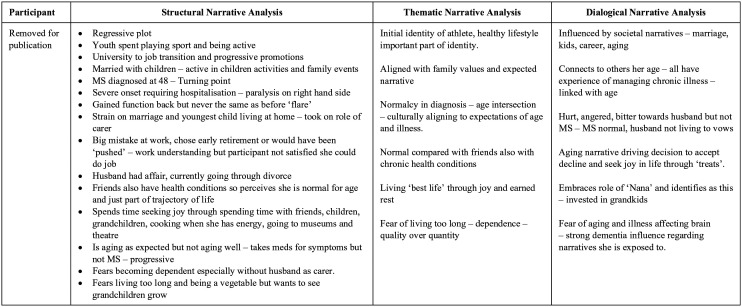
Example of pluralistic narrative analysis organization.

Structural narrative analysis focuses on the plot, organization, and distinct structures that scaffold the story and hold it together (Smith, 2016). As a first lens, this allowed us to identify different narrative types, important timepoints, and story plots of aging with MS. Thus, structural narrative analysis allowed us to build a template for different aging with MS stories. This analytical lens has been used previously in MS to explore the evolving perception of exercise over time (E. V. Richardson et al., 2018). To do this analysis, we followed the guide of Smith (2016). We first engaged in narrative indwelling whereby we conducted, read, and reread transcripts to immerse ourselves in the data. At this point, we started writing about the stories within the data and continued this throughout the analytic process, as writing is analysis (L. Richardson, 2000). We read each transcript specifically noting how the stories were shaped, the direction/plot of the story, reflections of participants, tone and changes of tone, conflicts, characters, attitudes and emotions; effectively building the structure of narratives. At this stage, a separate word document was created to combine analytical findings. Each participant was assigned a row and three columns that represented the findings for each participant regarding structural, thematic, and dialogical narrative analysis. An example is provided in Figure 2.

##### Thematic narrative analysis

Once each transcript was analyzed structurally, we then moved to thematic narrative analysis and the “whats” of the story (content). This analysis allowed us to identify central themes of participants’ stories, patterns, relationships, and discourses to build around the narrative structure and provide more details and context (Riessman, 2008). To do this, we again engaged in analytical writing (L. Richardson, 2000) by reading each transcript and asking questions such as “what are common themes in this story?” “What relationships are mentioned?” and “What is the common thread throughout?” We also highlighted emotive words that were perceived to reflect the essence of an experience (e.g., helplessness, empowerment) to ensure this was captured in the final stories. At this stage, we also began interpreting themes in relation to previous research and theory (Riessman, 2008) that would be drawn upon later when we wrote the stories. For example, a key theme was how participants gained a sense of how “well” they were by assessing and comparing themselves with peers. This has been highlighted in previous research exploring aging well with MS (Finlayson et al., 2005). We then explored this further through social comparison theory (Festinger, 1954), and how participants compared themselves with others in an either upward or downward trajectory, and how this shaped participants’ sensemaking regarding aging with MS. Themes of each transcript were transposed onto the thematic narrative analysis column aligning with the relevant participant. Once this was complete for all transcripts, we moved to a DNA lens.

##### DNA

DNA “studies the mirroring between what is told in the story—the story’s content—and what happens as a result of telling the story—it’s effects.” (Frank, 2010, pp. 71–72). By doing a DNA, we could again expand stories to capture emotive, affective experiences, show why participants told specific stories, and the impact these stories had on the person. To do this analysis, we drew upon the heuristic guide of Frank (2010). By engaging in dialogical questions focusing on resources and circulation, we could again explore the “whats” and “hows” of stories (first explored through narrative and thematic narrative analysis) for rigor. Furthermore, by exploring the effects of stories, we expanded the building blocks of the narratives to include how stories shaped individuals’ sense of self, how stories (dis)connected individuals to and from others, cultural resources used to shape how participants made sense of aging with MS, and how stories had an impact on feelings and emotions of participants. For example, participants felt very disconnected to peers that told different narratives of aging. The effect of this was almost a judgmental or pitying view of “others” and we sought to present this in our stories. Furthermore, someone viewing aging as normalizing their embodied experience may have made sense of this through a Western aging narrative (Gullette, 2004). By identifying this resource, we could explore why this particular narrative was chosen by a participant to make sense of their aging experiences, and the impact this had on how they chose to manage aging with MS. DNA findings were transposed onto the appropriate column. Thereafter, we moved to the next stage of constructing the creative nonfictions.

#### Building a typology

A narrative typology is a set of narratives that constitute various types, and allow for naming differences between experiences (Frank, 2013). By building a typology of narratives, we could more readily show two very different stories of aging with MS. We identified storied differences by grouping together similar stories that were crafted from the initial pluralistic analysis. Two plot trajectories were noted: a progressive narrative trajectory whereby participants told a life story that improved over time and a regressive narrative trajectory whereby participants told a life story that declined over time.

We designed a basic plot structure comprising a past, present, and future perception of aging with MS as a base for crafting the stories. The progressive narrative followed a basic plot of

I was diagnosed with MS at a young age at a time where no treatments were available, and I expected to die young. Today, I view aging with MS as a blessing as I did not expect to experience this. Though I experience physical and cognitive symptoms, these experiences seem normal at this stage of life, and I have found effective lifestyle strategies to manage these. I am positive for my future with MS, but worried about age related illnesses that might impact my quality of life.

Alternatively, the regressive plot followed a structure of

I lived a “normal” early and mid-adulthood without MS. I was diagnosed with MS later in life and have experienced a steady physical and cognitive decline. Though I have MS, everyone my age has a chronic condition and I therefore perceive I am normal for my age so my MS does not require extensive intervention. I intend to enjoy my life as much as possible and have earnt rest and retirement after a life of hard work. I do worry about continual decline, and I do not want to live so long that I lose my independence and quality of life.

With the basic typology established, we then built the narratives further by incorporating themes, detailing plot lines, beginning to craft the story, and incorporating theory.

### Building the Stories

The four different methods of building the stories were used iteratively, and we alternated between a story analyst and storyteller lens.

#### Thematic maps

Key themes for each story were crafted via narrative thematic analysis. The “Kicking and Screaming” narrative was shaped by themes of identity work, liminality, and resistance. Alternatively, the “Gracefully Conceding” narrative was shaped by themes of intersection, cultural alignment, and peer comparison. We sought to thread these themes throughout the characters’ stories to artistically show specific experiences and meanings of aging. We further drew upon different theories (detailed below) related to each theme to add depth to the stories and theorize the data.

#### Story capture

With a basic outline and initial themes noted, we maneuvered to a storyteller standpoint to “write” the story. We engaged heavily in story capturing (Mueller, 2019) where we copied large parts of participant stories directly from interview transcripts that reflected a certain plot point or theme in a rich and evocative way. We then transposed these data excerpts into the story itself and began restructuring the order in a way that encouraged flow. For example, one participant told a negative story of being diagnosed with MS in 1980. Many participants stated the diagnosis experience at this time was inherently negative, but one particular participant told a very compelling, meaningful, and detail-oriented story regarding her interaction with the neurologist. We thus copied her testimony and positioned this as the beginning of the “Kicking and Screaming” narrative:

Interviewer: Can you tell me more about what it was like being diagnosed at that time? Participant: It was traumatizing, truly. He marched in, never seen him before, sits downs and says “well you know what you have right?” I told him no and then he said “oh, well why do you think you’re seeing me?” I was so angry. I told him I didn’t know. I thought I was seeing my other doctor. He said “but you know you have MS?” That was the first time someone told me. He then went on almost a spiel that he rattles off, kind of like when you hear terms and conditions. “You have some lesions on your brain, spine, and your optic nerve though not too many at this point.” He told me not to research MS as I wouldn’t find anything to comfort me. As far as a cure, there wasn’t one. And as far as medications are concerned, we won’t see them in my lifetime. MS is a disease that will slowly attack my physical and mental capabilities. I would get worse physically and cognitively worse to the point that I would need long term care. I would be in a wheelchair within a year, bed bound in 5 and perhaps in a nursing home, and most people can only expect to live 10 years or so once it appears. He said, “you probably had multiple sclerosis starting 4 years ago with your first episode of weakness.” I just felt sick. Then, and this just was a punch in the gut; he said I was lucky! He said “You’re lucky you know? You’ve worked, married, had your children so your life has been fulfilling. A lot of people don’t get that so count yourself blessed. I would however prepare the family as it’s hardest on them, especially your husband, so hard he may leave you. That happens to a lot of my patients; it’s just too much for the spouse to bear.” I couldn’t believe what I was hearing. Forget me, he’s more concerned for my husband!?

We used this conversation as a base for the “Kicking and Screaming” diagnosis scene and drew upon other participant’ testimonies to create layers of emotion and internal dialogue to the character in this story. We continued this process until we had strong, evocative stories that represented each theme and plot point.

#### Outline plot

Although a basic plot line was established through narrative structural analysis, the additional building blocks of themes and story capturing allowed us to expand the plot, ensure flow, and add layers of emotive textures for the story to get under people’s skin. At this stage, we decided to structure each story through three chapters that represented the “past, present, and future” perceptions of aging with MS. For each chapter, we crafted a scene, (e.g., neurologist office, the character’s home), necessary supporting characters, and turning points and incorporated important empirical details about living with MS, aging with MS, and other important informational pieces we wished to share through the stories. For example, we wanted to share information about how some participants in the “Kicking and Screaming” narrative perceived they aged well. By outlining a scene where “Jane” is asked to share how she ages well, we could portray this information in a storied, yet empirically robust, way rather than a list that may not translate knowledge meaningfully.

#### Theorizing

A good creative nonfiction is theoretical in itself (Smith, 2013). We therefore utilized various theoretical and conceptual frameworks from sociology of health and illness, narrative gerontology, and critical gerontology to enhance our stories. The theories we used were guided by the respective themes of each story. For example, in the “Kicking and Screaming” narrative, themes of identity work, liminality, and resistance held this story together. Changing and reconstructing conceptions of the self is a theme identified across chronic illness (e.g., Charmaz, 1991) and indeed within the context of MS itself (Toombs, 1995). Thus, self-hood helped us expand how Jane renegotiated a sense of self as she aged. Furthermore, the liminal self (van Gennep, 1960) has been effectively used to explore transitions of the self after an MS diagnosis (Strickland et al., 2017). Liminality is a framework that encompasses “rites of passages” through three different periods: preliminal (rites of separation), liminal (rites of transition), and postliminal (rites of reincorporation). In Jane’s story, we incorporated all three of these stages, and how her identities at these times shaped how she made sense of aging with MS. Furthermore, we show how Jane resists expectations of having MS, and aging with MS, in various ways by living her own story. There has been increasing focus on telling “counter narratives” (Nelson & Lindemann, 2001) of aging that challenge the dominant Western narrative of decline (Gullette, 2004) and taken-for-granted assumptions of aging (Zeilig, 2011). By telling stories of active aging (Phoenix & Smith, 2011), for example, there is resistance to negative aging narratives and an empowering movement to tell one’s own story. We show a potential counter narrative to aging with MS through Jane’s story.

The “Gracefully Conceding” narrative tells a very different story and was shaped by themes of intersection, cultural alignment, and peer comparison. As such, we theorized this narrative further by drawing upon intersection theory, narrative gerontology, and social comparison theory. Intersectional theory explores how different identities intersect to craft a sense of self and understanding of phenomena (Crenshaw, 2017). In this case, “Jim” being diagnosed at an older age with a chronic illness intersects to shape how he makes sense of aging with MS. That is, Jim is exposed to different cultural narratives than Jane upon his diagnosis. Being in his late 50s, he already relates to the dominant Western narrative of decline, and perceived diagnosis of a chronic condition as merely a normal aging process. This is further cemented through peer comparison. Peer comparison is an established way by which people aging with MS try to make sense of, and adapt to, a life with this illness (Dilorenzo et al., 2008). Social comparison theory stipulates that people determine their own social position and personal worth based on how they compare with others that are similar (Festinger, 1954). By comparing himself with peers who also have chronic conditions, we show how and why Jim perceives that he does not need to intervene to manage his MS. Once we were content with our detailed plot, themes, stories and theories, we moved to a storyteller lens by writing and refining the stories.

#### Writing and refining

In considering the final presentation of the stories, we reflected on what language, dialogue, scene creation, characters, and narrative overviews were required to create believable interactions and a meaningful knowledge translation of aging with MS. We therefore utilized numerous fictional literary techniques to link the stories, plot, and theory together in a storied fashion. To show how we created the nonfictions, we provide a “Data Source” column adjacent to the stories, akin to the structure of Hall (2020). Here, we show various stages of the process and why these particular stories were told.

After completing a first draft of both stories, we shared these with colleagues who acted as critical friends by challenging the stories, acting as a theoretical sounding board, and offering different ways of interpreting and telling stories of aging with MS. We took these critiques on board and refined the stories and manuscript as a whole. There were further extensive cycles of review and refinement through the peer review process. The journal reviewers provided detailed, thoughtful critiques that allowed for a much stronger manuscript and evocative stories. We now present out finalized narratives of aging with MS which we present in the Supplemental File attached to this article (see Supplemental File).

## Discussion and Recommendations

The purpose of this research was to engage in a creative, transformative methodology to show different stories of aging with MS. We outline tentative recommendations for how to use the stories as knowledge translation devices in practice. Within our recommendations, we critique our own work by highlighting limitations and suggestions for future work.

We recommend three particular uses of the stories to (a) validate the various experiences depicted in the narrative, (b) show how and why persons with MS make sense of aging differently, and (c) highlight various fears and concerns persons with MS may have about aging with MS

### Validating Experiences

Stories have the ability to validate an individual’s experience as they see themselves represented in text (Frank, 2013). That is, an individual may feel connected and empowered with this representation, and take on knowledge that is shared through this medium (Stake, 1995). We told particular stories of “Kicking and Screaming” and “Gracefully Conceding, as these were dominant throughout participants’ testimony. We therefore perceived that either narrative may be lived by many persons with MS such that they can connect to and feel validated in their aging with MS experiences.

A further strength of these stories is expanding the current cultural menu (McAdams, 2006) of aging with MS narratives. In Jane’s story, she discussed frustration and worry about only a tragic narrative of aging with MS being available to make sense of this experience. By promoting a progressive “Kicking and Screaming” narrative, we show a different, more positive narrative whereby aging with MS has numerous benefits and perhaps an enhanced quality of life. Such an addition to aging with MS stories may act as a positive narrative map for those concerned about aging with MS. Narrative maps are guides that provide orientation, information, and advice to newcomers regarding how to navigate an unknown experience (Pollner & Stein, 1996). Such a concept has been used effectively regarding counter narratives to “aging is decline” (e.g., Phoenix & Sparkes, 2006), and can be effective regarding aging with MS. By showing Jane’s story including how she “ages well” and manages different symptoms, this may inform persons with MS that there are affirming ways to age with MS, with some suggestions of how to do so. Furthermore, we show a way through Jane’s management of her symptoms that she was able to craft a sense of self-hood (Maietta, 2021) that was more empowering to her rather than a “sick role” (Cheshire et al., 2021) which dominate social narratives of MS. Amplifying and promoting this narrative to individuals who mirror the MS trajectory of Jane (i.e., mild to moderate symptoms, relatively stable relapses) may address concerns regarding solely negative portrayals of aging with MS being available. As such, this experience may not be something to be feared. Furthermore, the character of Jane can act as a role model for individuals seeking information and a way to “age well” with MS.

Equally, some individuals may align to Jim’s narrative whereby they experience more severe symptoms that have affected their plans for later life. Within Jim’s story, we show how he made sense of aging with MS through comparing himself with others and how he aligned to Western cultural narratives of aging. The results of these comparisons validated for Jim that the symptoms and progressive dysfunction he experienced were normal for this stage of life. Shaping meaning and experiences of aging this way has been highlighted in the literature as underlying social, cultural, and individual processes intersect to craft an explanation of this phenomenon (Moody, 2008). Comparing with peers, social narratives and Jim’s own embodied experiences are apparent in his story, and may be common for others that mirror Jim’s MS journey. We posit that this may be a way to cope with a difficult embodied experience as, when faced with uncertainty in chronic illness, we turn to narrative to make sense of our lived experiences (Bury, 1982). In this case, narratives of decline made sense and positioned Jim as normal for his age. We note that “Gracefully Conceding” is a more difficult story for people to read as it follows a regressive plot. We did not write this for the intention to perpetuate a negative story, nor to upset anyone reading this, but to reflect the lived, embodied experiences of some persons aging with MS.

The topic of aging with MS is difficult for health care providers, and communicating health care information to persons with MS is a continually pressured expectation as new phenomena (such as aging) add to the list of areas that must be covered. The patient–provider relationship is very important with regard to receiving information about MS as health care providers are often the first port of call regarding questions patients have about MS (Learmonth et al., 2017). Within aging, however, evidence-based information is still lacking and many uncertainties remain (Hua et al., 2019). This is a difficult situation for health care providers as they treat and inform patients to the best of their ability. Furthermore, the demands of a health care provider’s time and effort to cover so many different aspects of MS management (e.g., disease modifying therapies, symptom changes, and lifestyle management) as well as now learn about aging are overwhelming (E.V. Richardson et al., 2020). We hope that health care providers reading these stories can recognize and appreciate (a) the great advancements that have been made regarding MS knowledge and the patient–provider relationship and (b) the challenging situation they are in when treating patients aging with MS.

We note, however, that these narratives should be used with caution as they capture only two stories of aging with MS. Creative nonfiction cannot capture each person’s lived narrative, nor was it our goal to do so. We therefore encourage practitioners, family and friends, and persons with MS to remember these stories are not absolutes and alternative stories may have been crafted with different participants and researchers. We encourage future researchers to consider the power of narrative regarding validating experiences of aging with MS, caring for someone aging with MS, and treating patients aging with MS, and continue to expand the cultural menu of aging with MS narratives that can help individuals make sense of this phenomenon. A further limitation regarding the transmission of these stories is that they are told only from the person with MS point of view; we did not interview any health care providers; thus, health care providers were secondary characters and we did not present an insight regarding their thoughts and emotions. We encourage future researchers to explore the difficulties, practicalities, and requirements of health care providers regarding treating patients aging with MS, and appropriate supports to ensure these individuals’ health and well-being.

### Showing Persons with MS Experiences of Aging

Although there is increasing research exploring what aging with MS may look like and the different trajectories this may take, there is still a gap regarding why and how people make sense and meaning of aging in different ways. We addressed this gap by showing two different narratives of aging with MS, incorporating why Jane and Jim told such different stories. We did this in the hope of translating knowledge about aging with MS to an understandable medium that is accessible to the majority of audiences. Creative practices to translate empirical knowledge and academic language have been effectively accomplished about spinal cord injury rehabilitation (e.g., Smith et al., 2015), and social relationships doing physical activity with a physical impairment (Javorina et al., 2020). We add to this knowledge translation effort by sharing information about aging with MS. For example, we used the composite character of Dr Campbell to share knowledge regarding current understandings of the influence age of diagnosis plays related to progression of dysfunction, different therapeutic strategies to manage MS symptoms, and the advancements that have been made regarding aging with MS through Jane’s conversations with friends and her husband. Through stories, we hope that persons with MS, and friends and family of persons with MS, may be better informed about aging and MS.

Another objective we had regarding showing two different, but equally valid, experiences of aging with MS is to potentially reduce the disconnect between individuals who align to each narrative. As stated in the “Data Source” commentary, there was division to the point of dislike of individuals who lived a different narrative. We hope, by showing in detail why persons tell different stories of MS, this may enhance understanding and empathy of those who age with MS differently.

Research has highlighted the importance of strategies such as exercise, nutrition, and mindfulness for living well while aging with MS (e.g., Ploughman et al., 2012); however, some persons aging with MS choose not to engage in such strategies. We sought to illustrate why persons chose to engage, or not engage, in lifestyle strategies through the characters of Jane and Jim. We show these stories not to promote a preferred narrative, but to highlight from participant testimony reasons why persons may choose to take an active role in managing MS or not. Our intention was to show health care providers potential underlying reasons for the different ways persons with MS age that may not arise from appointment conversations. In this way, health care providers may consider other ways to provide appropriate support.

We note again that these are two dualist narratives that do not represent the entire aging with MS experience, nor all reasons that persons make sense of aging with MS differently. Furthermore, these stories are culturally specific to the United States, and different stories may be told in other cultures with different narratives of aging, MS, and MS treatments. We encourage researchers in other cultures to explore the potential of narrative sensemaking to share stories of aging with MS for the benefit of health care providers, and persons with MS.

### Fears and Concerns of Aging with MS

Narratives provide a lens to illuminate individual-level fears, concerns, inner thoughts, and perceptions (Smith & Sparkes, 2008b). In the case of the creative nonfictions, we wished to highlight the various fears and concerns participants had regarding aging with MS. Jane and Jim discussed similar fears regarding losing their independence and quality of life, age-related illness such as dementia, and the general unknown about what the future holds. We did this for the following reasons. First, we again wished to validate readers with MS fears regarding aging with MS and highlight that they are not alone in having these thoughts. Second, we wished to highlight to health care providers potential concerns patients with MS may have that they forget or struggle to articulate during appointments because of stress, brain fog, or the need to focus on a particular symptom that takes up appointment time. Third, we hope that by illuminating these common fears, health care providers may be aware of these potentially unspoken concerns and address these in appointments. Fourth, we hope these fears addressed by participants may encourage foundations to produce or direct persons to informational resources addressing these concerns, and that researchers may be encouraged to address these questions in their future endeavors.

## Conclusion

The two creative nonfictions in this article show two different, but equally valid, stories of aging with MS. This novel approach provides a meaningful way to show how persons aging with MS make sense and meaning of this lived experience, and to translate knowledge about this phenomenon to health care providers and persons with MS. These stories are but a starting point for crafting a larger cultural menu from which persons aging with MS can draw upon.

## Supplemental Material

sj-pdf-1-qhr-10.1177_10497323211009864 – Supplemental material for “Kicking and Screaming” or “Gracefully Conceding”: Creative Nonfiction Stories of Aging With Multiple Sclerosis Click here for additional data file. Supplemental material, sj-pdf-1-qhr-10.1177_10497323211009864 for “Kicking and Screaming” or “Gracefully Conceding”: Creative Nonfiction Stories of Aging With Multiple Sclerosis by Emma V. Richardson and Robert W. Motl in Qualitative Health Research
