# Supplementary material for: “Kicking and Screaming” or “Gracefully Conceding”: Creative Nonfiction Stories of Aging With Multiple Sclerosis
Source: Qual Health Res. 2021 Apr 30;31(10):1861–74. doi: 10.1177/10497323211009864 (PMC8446897; doi:10.1177/10497323211009864)
Supplement: sj-pdf-1-qhr-10.1177_10497323211009864 – Supplemental material for “Kicking and Screaming” or “Gracefully Conceding”: Creative Nonfiction Stories of Aging With Multiple Sclerosis [file sj-pdf-1-qhr-10.1177_10497323211009864.pdf]

Interview Guide

Questions on Aging and MS

1. Grand Tour: Please tell me about yourself
    a. Age, MS diagnosis, geographic area, job, children, pets, partners
2. Please tell me about your life up until this point
    a. What was your life like before your diagnosis?
3. What impact did an MS diagnosis have on you?
    a. Please tell me about your diagnosis experience.
4. What impact does MS have on you now?
5. How has your MS changed over time?
6. Could you please paint a picture of what your life looks like right now?
7. Describe for me what it's like having MS and experiencing aging.
    a. What's changed in your experiences and perceptions from being younger with MS and being older with MS
8. How do you compare to your peers?

Questions on Wellness

9. What do you do to feel good?
10. What give you joy?
11. What makes you happy?
12. What helps/ facilitates your ability to do these things that make you happy?
    a. What stops you?
13. Can you tell me a story about when you felt 'well'
    a. What are you doing? What are you feeling, who is there?
14. What would an ideal wellness situation look like?
15. What does wellness mean to you?
16. What are key components of wellness?
    a. Physical, social, spiritual/ religious – key focus
17. What aspects of life make you feel well?
18. How does wellness incorporate into your life?
19. What impacts your wellness positively?
20. What impacts your wellness negatively?
21. What are your perceptions for the future?
22. Anything I've missed/ you would like to add?
